# Novel perceptions and insights into the rare hematologic malignancy of acute megakaryocytic leukemia: a multicenter clinical retrospective study

**DOI:** 10.3389/fmed.2025.1574132

**Published:** 2025-06-06

**Authors:** Yuan Liu, Yanquan Liu, Xiaojun Chen, Yue Yin, Zhenyuan Xu, Jiachen Xie, Jianzhen Shen, He Huang, Huidong Guo

**Affiliations:** ^1^The First Affiliated Hospital of Gannan Medical University, Ganzhou, China; ^2^Key Laboratory on Leukemia of Jiangxi Provincial Health Commission, Department of Hematology, Ganzhou Hospital Affiliated to Nanchang University, Ganzhou People’s Hospital, Ganzhou, China; ^3^Department of Hematology, The Affiliated Hospital of Putian University, Putian, China; ^4^Department of Hematology, Fujian Medical University Union Hospital, Fuzhou, China; ^5^Department of Pathology, The First Affiliated Hospital of Gannan Medical University, Ganzhou, China

**Keywords:** acute megakaryocytic leukemia, proto-megakaryocyte, differential diagnosis, clinical features, prognosis

## Abstract

**Objective:**

Acute megakaryocytic leukemia (AMKL) constitutes a rare subtype of acute myeloid leukemia in clinical practice and exhibits a high degree of heterogeneity. This study endeavors to explore the clinical manifestations, diagnosis, treatment, and prognosis of AMKL, offering novel perspectives for both basic and clinical investigations of rare myeloid tumors in the fields of oncology and hematology.

**Methods:**

The clinical data of 23 patients with AMKL admitted to the Fujian Medical University Union Hospital, the Affiliated Hospital of Putian University, and the First Affiliated Hospital of Gannan Medical University from January 2014 to July 2024 were retrospectively analyzed. The clinical characteristics, diagnosis and differential diagnosis, treatment, and prognosis of AMKL patients were examined. Additionally, the latest literature in the PubMed database was retrieved for review and discussion regarding the research advancements of AMKL and its diagnosis and treatment.

**Results:**

A total of 23 patients with AMKL were encompassed in this study, the clinical manifestations of all patients were predominantly hematological non-specific symptoms, such as anemia, bleeding, infection, and invasive swelling or occupation of tissues and organs. All patients underwent bone marrow puncture biopsy, cytochemical staining of bone marrow cells of AMKL patients demonstrated that the staining of POX, NAS-DCE, and hot brine test were negative, however, the PAS staining, *α*-NAE staining and NaF inhibition test were positive. Except for 2 patients who were not detected by flow immunotyping, cytogenetics and molecular biology, the remaining 21 patients were detected accordingly, and megakaryocyte antigens (CD41, CD42, CD61) were expressed in these 21 patients with AMKL, accompanied by certain cytogenetic or molecular biological abnormalities. There were two patients forsook treatment in our study, and remaining 21 patients who underwent clinical treatment measures, 1 patient (4.76%) died after 1 course of chemotherapy, 3 patients (14.29%) succumbed to severe infection occasioned by bone marrow suppression after 2 courses of chemotherapy, and 7 patients (33.33%) achieved CR after 1 course of chemotherapy, 4 patients (19.05%) attained CR after 2 courses of chemotherapy, and 6 patients (28.57%) failed to achieve remission (NR) after 2 courses of induction chemotherapy. Correspondingly, a total of 6 patients received allogeneic hematopoietic stem cell transplantation (HSCT) in this study, among which 3 patients received HSCT after CR in the first induction chemotherapy, 1 patient received HSCT after CR in the second round of induction chemotherapy, and 2 patients with NR after induction chemotherapy underwent HSCT. We conducted follow-up until July 31, 2024 and discovered that among the 17 patients who received complete and standardized treatment and survived, 3 (17.65%) patients were lost to follow-up and 8 (47.06%) patients perished within 2 years due to treatment failure attributed to disease progression, recurrence, and uncontrollable disease. The remaining 6 patients (35.29%) are still alive at present and have not experienced disease progression or recurrence. The median follow-up period was 33.5 months (ranging from 4.5 to 76 months) as of July 31, 2024, the results of survival analysis indicate: the OS and EFS of AMKL patients treated with chemotherapy alone were inferior to those treated with chemotherapy combined with HSCT (all *p* < 0.05). Additionally, AMKL patients with severely abnormal cytogenetic test results had poorer OS and EFS (all *p* < 0.05). Concurrently, the OS and EFS of AMKL patients who achieved CR after 2 courses of induction chemotherapy were significantly superior to those of AMKL patients who did not achieve CR (all *p* < 0.05).

**Conclusion:**

AMKL is infrequent in clinical practice, features a poor prognosis, lacks specificity in clinical manifestations, and is prone to misdiagnosis or omission. Clinical trials of new drugs should be prioritized, while close monitoring of measurable residual disease (MRD) should be implemented. Patients with AMKL might benefit from induced remission chemotherapy combined with novel targeted therapy. Hematopoietic stem cell transplantation should be carried out as soon as possible after the first CR induced by standard chemotherapy to optimize the prognosis.

## Introduction

Acute megakaryocytic leukemia (AMKL) constitutes a rare subtype of acute myeloid leukemia and is characterized by a poor prognosis. In accordance with the French, American, and British classification scheme for leukemia (FAB), AMKL is categorized as AML-M7 and is frequently associated with myelofibrosis (1). Based on the attributes of patients with AMKL, AMKL is typically subdivided into three clusters: AMKL in children with Down syndrome (DS), AMKL in children without Down syndrome, and AMKL in adults without Down syndrome, which might be related to specific and distinct pathogenic mechanisms and have dissimilar prognoses (2). DS-AMKL patients typically present with trisomy 21 (T21) and GATA1 mutations, accompanied by mutations in chromatin regulators (such as subunits of cohesive proteins and EZH2) or signaling molecules (such as molecules within the JAK/STAT and RAS pathways). Conversely, DS-AMKL patients tend to have a relatively favorable prognosis (3–5). In contrast, the pathogenesis of non-DS-AMKL in adults remains ambiguous, and the prognosis is poorer, with an overall survival of less than 1 year (6).

Due to the low incidence and clinical rarity of AMKL, as well as the high heterogeneity of the disease itself, AMKL is prone to missed diagnoses and misdiagnoses in clinical practice. Correspondingly, at present, the academic community still focuses on individual cases or very few single-center retrospective studies, and research on its pathogenic mechanism, clinical diagnosis, and treatment is relatively simplistic and restricted. To this end, this study retrospectively analyzed the clinical data of 23 patients with AMKL from 3 large medical centers in southern China and conducted a retrospective analysis and review of the literature with the aim of enhancing the understanding of AMKL in the fields of oncology and hematology.

## Materials and methods

### Research object

**Figure 1:**
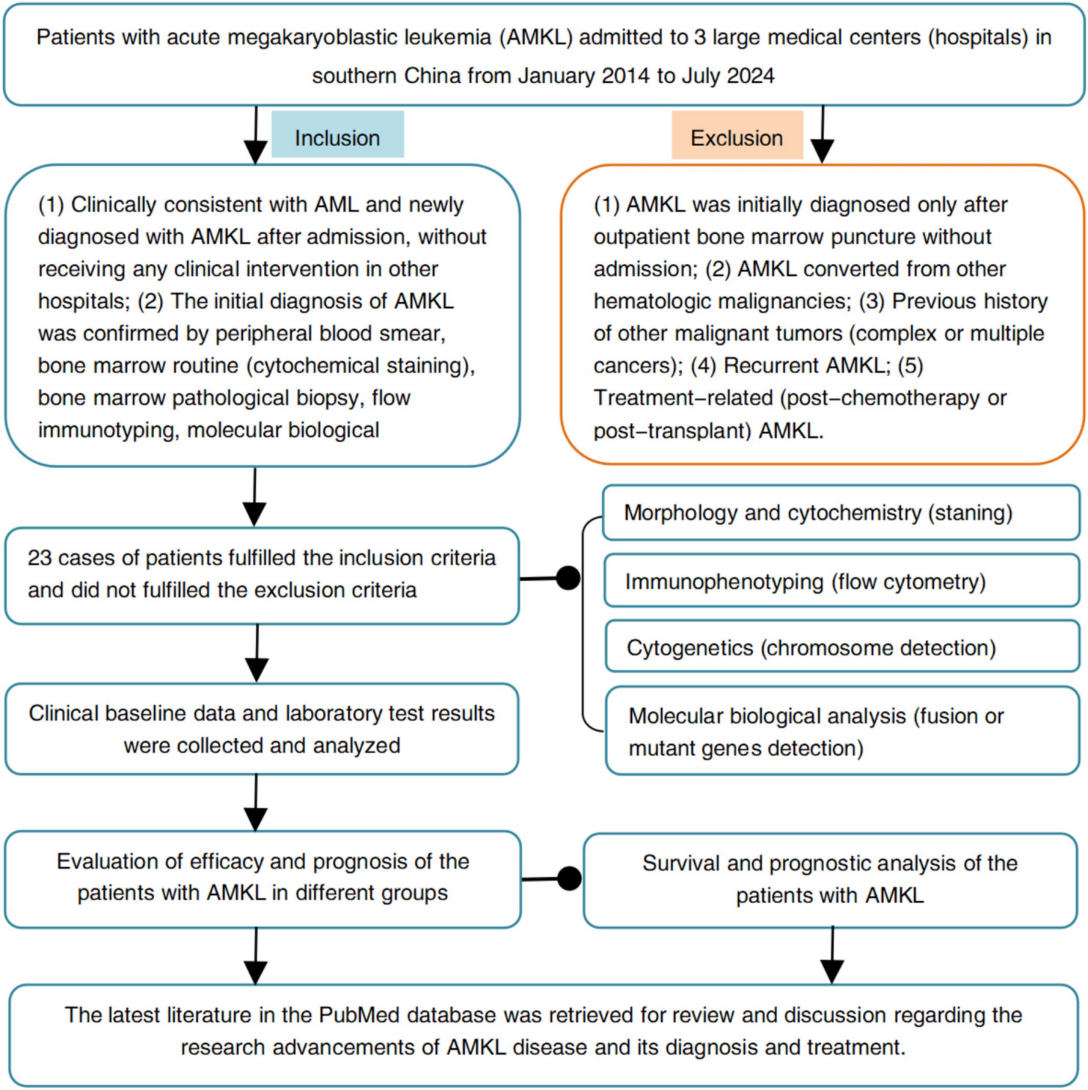
Flow chart of study design and screening sources.

The clinical data of 23 patients with AMKL admitted to three large medical centers in southern China, namely the Fujian Medical University Union Hospital, the Affiliated Hospital of Putian University and the First Affiliated Hospital of Gannan Medical University from January 2014 to July 2024, were retrospectively analyzed. The diagnostic criteria for AMKL refer to the WHO classification diagnostic criteria (7–9), that is, bone marrow original cells ≥20%, of which ≥50% originated from the megakaryocytic lineage; and the original cells express CD41, CD42, CD61 or VII factors. The flow chart of the design and screening sources for this study is shown in Figure 1.

### Research method

The inpatient diagnosis, treatment and prognosis information of enrolled patients were checked through the electronic medical record inpatient system of our hospitals, and the patients and their families were contacted by telephone for follow-up. Meanwhile, relevant materials such as clinical and laboratory examination results of patients were collected and sorted out. The above work has been approved by the Medical Ethics Committee of our hospitals, and carried out with the informed consent of patients and their families.

### Inclusion and exclusion criteria

Inclusion criteria: (1) Clinically consistent with AML and newly diagnosed with AMKL after admission, without receiving any clinical intervention in other hospitals; (2) The initial diagnosis of AMKL was confirmed by peripheral blood smear, bone marrow routine (cytochemical staining), bone marrow biopsy, flow immunotyping, molecular biological examination and other classical diagnostic methods of hematological diseases.

Exclusion criteria: (1) AMKL was initially diagnosed only after outpatient bone marrow puncture without admission; (2) AMKL converted from other hematologic malignancies; (3) Previous history of other malignant tumors (complex or multiple cancers); (4) Recurrent AMKL; (5) Treatment-related (post-chemotherapy or post-transplant) AMKL.

### Morphologic assessment

All AMKL patients in our study underwent bone marrow puncture biopsy, bone marrow smear was examined through morphologic assessment, namely, cytochemical staining, encompassing Wright-Giemsa staining, Peroxidase (POX) staining, Periodic Acid-Schiff (PAS) staining, Naphthol AS-D chloroacetate esterase (NAS-DCE) staining, *α*-Naphthyl acetate esterase (α-NAE) staining, as well as NaF inhibition test and Hot brine test. And the bone marrow biopsy tissue was stained by immunohistochemistry. Bone marrow slides were reviewed for multilineage dysplasia and abnormal megakaryocyte maturation, and if available, reticulin and collagen stains were reviewed or performed to grade bone marrow fibrosis (MF-0 to MF-3) per standardized guidelines.

### Histological analysis and immunohistochemistry

Approximately 0.5 ~ 1 cm of bone marrow biopsy tissue was embedded in paraffin, and sectioned. These sections were subsequently stained with hematoxylin and eosin (H&E), dehydrated, and mounted for examination. Histopathological changes were evaluated and scored using an optical microscope. For the immunohistochemical process, the paraffin sections were deparaffinized in xylene, rehydrated in a graded alcohol series, and washed. Antigen retrieval was conducted in EDTA buffer (pH 9.0) using a microwave: medium power for 8 min, rest for 8 min, then medium-low for 7 min. After cooling, sections were washed with PBS (pH 7.4) and blocked with normal rabbit serum. They were incubated overnight at 4°C with anti-CD61 (Abcam), anti-MPO (Abcam), anti-CD34 (Abcam), anti-CD117 (Abcam), followed by goat anti-rabbit secondary antibody (Abcam) for 1 h at room temperature. Visualization was done with DAB, and sections were counterstained with hematoxylin. Slides were documented with an Aperio CT 6 scanner (Leica Microsystems, Germany) and analyzed using ImageJ (NIH, USA).

### Immunophenotyping

A six-color fluorescent direct immunoassay was used to detect the expression of surface antigens. More than 20% of antigen-expressing cells in immature myeloid cells were considered positive. For surface staining a 6-color protocol was used: 100 μL peripheral blood was incubated for 10 min with the following monoclonal antibodies: CD13-PE (BD Biosciences), CD33-APC, (BD Biosciences), CD34-FITC (BD Biosciences), anti-HLA-DR-APC-H7 (BD Biosciences), CD117-APC (BD Biosciences), CD56-PE-Cy7 (BD Biosciences), CD3-APC (BD Biosciences), CD4-PE-Cy7 (BD Biosciences), CD7-V450 (BD Biosciences), CD45-PerCP-Cy5.5 (BD Biosciences), CD61-FITC (BD Biosciences), CD11b-PE (BD Biosciences), CD41a-FITC (BD Biosciences), CD42b-PE (Beckman Coulter, Inc.) and CD36-FITC (Beckman Coulter, Inc.) at room temperature, then subsequently lysed for 10 min using 2 mL FACS-Lysing solution (BD Biosciences). After staining, the samples were washed with 2 m Cell Wash (BD Biosciences) and analyzed using a FacsCanto flow cytometer (BD Biosciences) by collecting 100,000 events. For analysis, the FacsDIVA software (BD Bioscience) was used. Blast cells were gated based on their side-scatter and dim CD45 characteristics. If the CD61 and CD42b were found to be positive from the peripheral blood, this suggested the patient had AMKL.

### Cytogenetic assessment

The karyotypes were analyzed by the G banding technique and described according to the ISCN 2009 standard (10). All cytogenetic results for this study were centrally reviewed and recorded using International System of Human Cytogenetic Nomenclature. The number of cytogenetic abnormalities and/or presence of any recurrent translocations were recorded.

### Molecular assessment

Aberrant genes detection on 3 mL of bone marrow specimens in EDTA were performed by the real-time polymerase chain reaction (qRT-PCR). Screening for multiple mutated genes specific to acute myeloid leukemia was performed as previously described (2, 11). RNA-sequencing was performed to identify fusion transcripts using total RNA extracted from patient samples using AllPrep DNA/RNA/miRNA Universal Kit (QIAGEN, Valencia), by the QIAcube system. The ribodepletion 2.0 protocol (British Columbia Genome Sciences Centre, Vancouver, BC) was employed to prepare the mRNA libraries with 75-bp strand-specific paired-end sequencing.

### Treatment plan (regimens)

Among the 23 patients with AMKL included in this study, 8 (34.78%) received the IA regimen (IDA + Ara-C); 4 cases (17.39%) were treated with azacytidine demethylation. 4 cases (17.39%) randomly contained DAE (DNR + Ara-C + Etoposide) or DAH (DNR + Ara-C + HHT); 3 cases (13.04%) were treated with the FLAG-IDA regimen (Flu + Ara-C + G-CSF + IDA) combined with intrathecal injection chemotherapy through lumbar puncture for prevention and treatment of central nervous system leukemia (CNSL). 2 cases (8.70%) received the decitabine + CAG regimen; The remaining 2 cases (8.70%) refused treatment after preliminary diagnosis of bone marrow morphology.

### Therapeutic effect evaluation

The evaluation and judgment of the efficacy of AMKL patients refer to the latest international standard for diagnosis and efficacy of hematological diseases. Evaluations of bone marrow aspirates were performed after the first and second inductions. The evaluation was conducted after each course of consolidation chemotherapy. The evaluation was conducted every 6 months during maintenance treatment until the end of chemotherapy. CR was defined as bone marrow with <5% blasts and evidence of the regeneration of normal hematopoietic cells; and minimal residual disease (MRD) ≤ 10^−4^. Relapse was defined as the presence of ≥5% blasts in the bone marrow or extramedullary relapse.

### Prognostic follow-up

In this study, the internal electronic inpatient record system of the hospital was used to follow up the indicators of the patients’ recent return to the hospital for re-examination and hospitalization treatment, and the follow-up was conducted by telephone contact. The last follow-up date was July 31, 2024, follow-up time range from 4.5 to 76 months. Overall survival (OS) was defined as the interval between the date of diagnosis and the onset of death or loss of follow-up (the end point of follow-up). Event-free survival (EFS) was defined as the interval between the date a patient started treatment with a standard regimen and the occurrence of a relapse due to any factor or a follow-up endpoint event.

### Statistical analysis

IBM SPSS 23.0 statistical software was used for statistical analysis of the collected clinical data. Chi-square test was used for comparison between the classification data groups, the Kaplan–Meier method was used for survival analysis, univariate and multivariate analysis of prognostic factors was carried out. Survival curves were drawn with GraphPad Prism 7.0 software. The significance level was set at *p* < 0.05.

## Results

### Clinical features

Among the 23 patients with AMKL encompassed in this study, 15 cases (65.22%) were male and 8 cases (34.78%) were female. There were 10 cases (43.48%) of pediatric patients (under 14 years old), with 7 males and 3 females, and a median age of 2.5 years (ranging from 11.5 months to 7 years old). Besides, there were 13 cases (56.52%) of adult patients, 8 males and 5 females, with a median age of 54.5 (ranging from 17 to 78) years. Given that AMKL is a rare disease, the sample size incorporated in this study was limited, and statistical analysis indicated that there was no statistical significance between the two groups (all *p* > 0.05). Hence, the 23 patients included were categorized into two groups, namely pediatric patients and adults, for classified analysis and presentation. The specific clinical characteristic parameters, such as clinical symptom indicators, peripheral blood indicators, and bone marrow examination, are presented in Table 1.

**Table 1:** Clinical features of the patients with AMKL.

Parameter	Pediatric patients (*n* = 10)	Adult patients (*n* = 13)
Age/years (range)	2.5 (11.5 months ~ 7 years)	54.5 (17 ~ 78)
Gender [*n* (%)]		
Male	7 (70)	8 (61.54)
Female	3 (30)	5 (38.46)
Clinical symptoms and manifestations [*n* (%)]		
Fever	7 (70)	9 (69.23)
Anemia	9 (90)	13 (100)
Bleeding	6 (60)	9 (69.23)
Hepatosplenomegaly	5 (50)	6 (46.15)
Lymph node enlargement	1 (10)	3 (23.08)
Laboratory indicators of peripheral blood examination		
WBC (×10^9^/L)	23.47 ± 18.31	15.68 ± 10.92
Hb (g/L)	73.92 ± 19.41	61.56 ± 14.78
PLT (×10^9^/L)	38.49 ± 23.62	51.08 ± 36.47
Aberrant elevated LDH	9 (90)	11 (84.62)
Bone marrow examination [*n* (%)]		
Bone marrow dry aspiration (tap)	6 (60)	10 (76.92)
Megakaryocyte antigens expression	10 (100)	13 (100)

WBC, white blood cell count, with the normal value being (4–10) × 10^9^/L; Hb, hemoglobin, the normal value for adult males is 120–160 g/L, for adult females 110–150 g/L, and for children 115–160 g/L; PLT, platelet count, the normal value is (100–300) × 10^9^/L; LDH, lactate dehydrogenase, the normal value is 109–245 U/L.

### Wright-Giemsa staining of bone marrow cells

**Figure 2:**
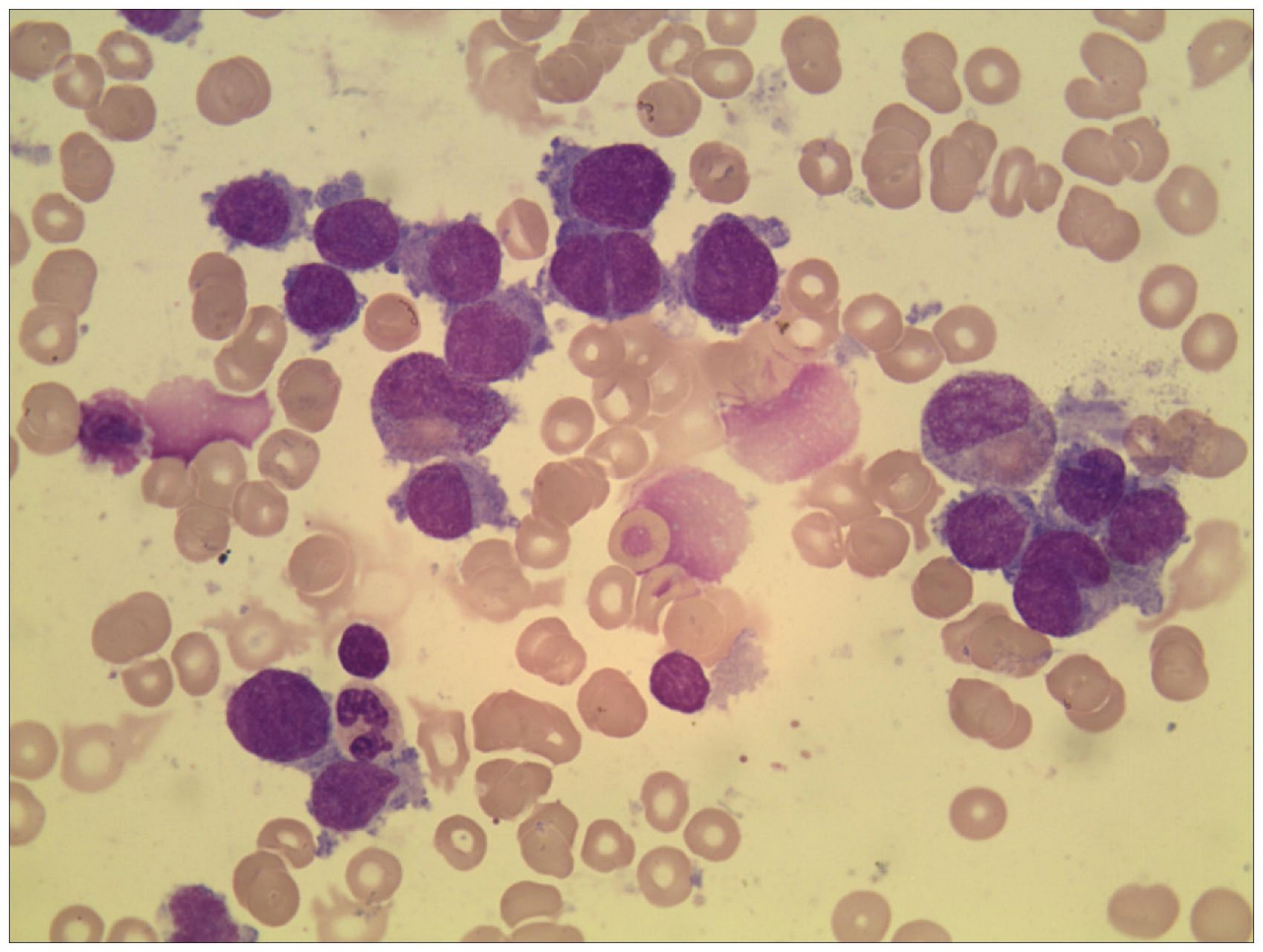
Wright-Giemsa staining results of bone marrow cells in AMKL patients (×400). In the figure, the shape of immature megakaryocytes was almost round, and the edge of the cell body could be seen as a cloud or flocculent process, accompanied by a few platelets.

All patients included in this study underwent bone marrow puncture. As depicted in Figure 2, the results of Wright-Giemsa staining of bone marrow cells indicated that patients with AMKL presented varying degrees of bone marrow hypercellularity. The bone marrow smears revealed immature megakaryocytes of diverse sizes, being round or quasi-round with uneven edges and featuring burr or cloud-like protrusions. The cytoplasm of these immature megakaryocytes was not abundant, showing a blue opaque and uneven coloration, with pseudopod protrusions and a few vacuoles or particles in some cases, as well as pits and distortions. The chromatin was fine or granular, but some nucleoli were not distinct, and aggregated platelets could be observed at the edges of some cells. Generally, more than 30% of promegakaryocytes were observed under the microscope, and the proliferation of erythroid and granular cells was relatively inhibited.

### Cytochemical staining of bone marrow cells

**Figure 3:**
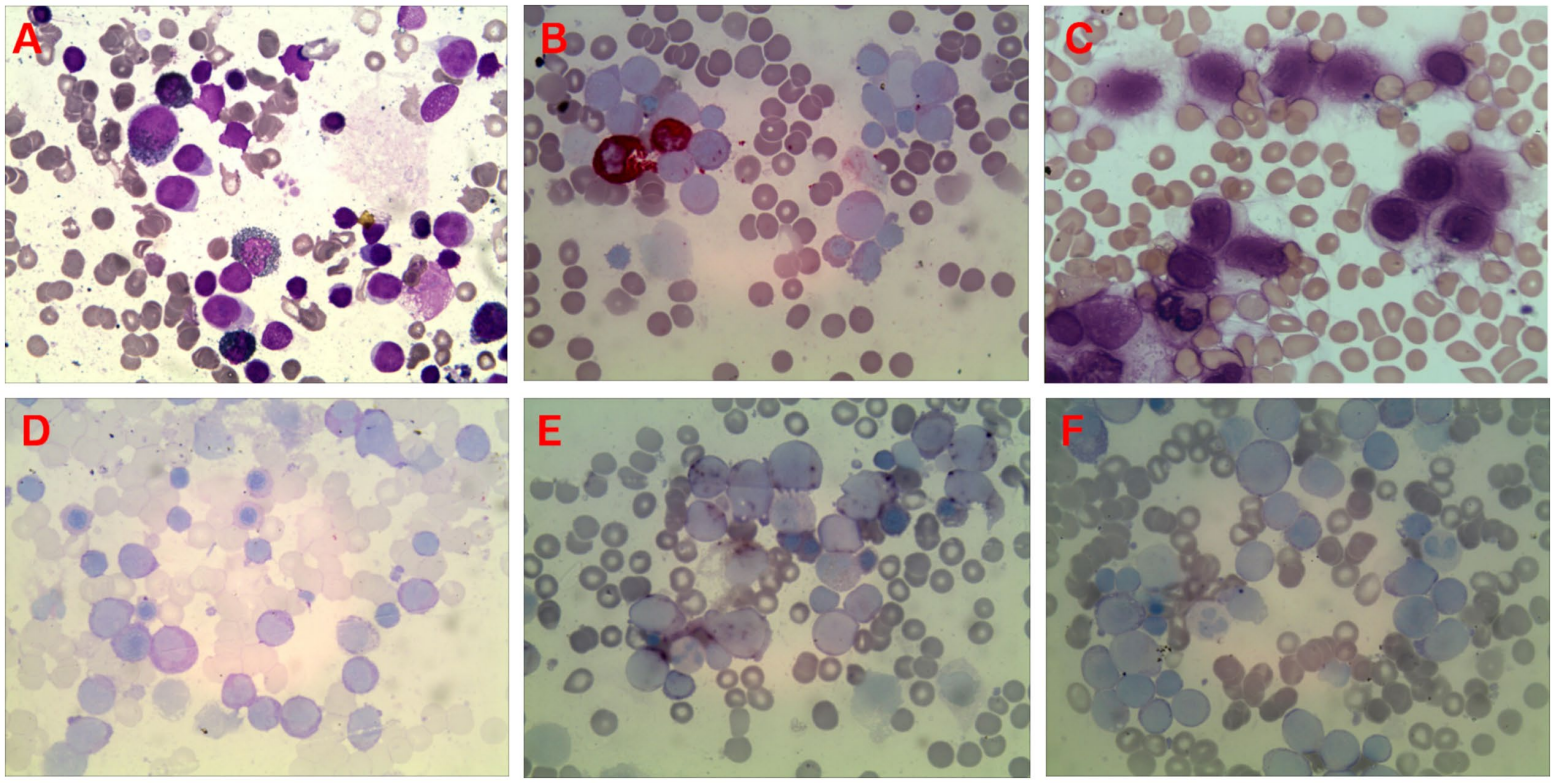
Microscopic image of bone marrow cytochemical staining of AMKL patients (all for ×400). **(A)** POX staining was negative; **(B)** NAS-DCE staining was negative; **(C)** The hot brine test was negative; **(D)** The original naive cells (immature megakaryocytes) were block-positive by PAS staining; **(E)** *α*-NAE staining was positive; **(F)** The NaF inhibition test showed that the original naive cells (immature megakaryocytes) were inhibited.

As shown in Figure 3, the cytochemical staining of bone marrow cells of AMKL patients demonstrated that the staining of POX, NAS-DCE, and hot brine test were negative. However, the PAS staining was positive in clumps, and *α*-NAE staining was positive and could be inhibited by the NaF inhibition test.

### Immunohistochemistry of bone marrow biopsy tissue

**Figure 4:**
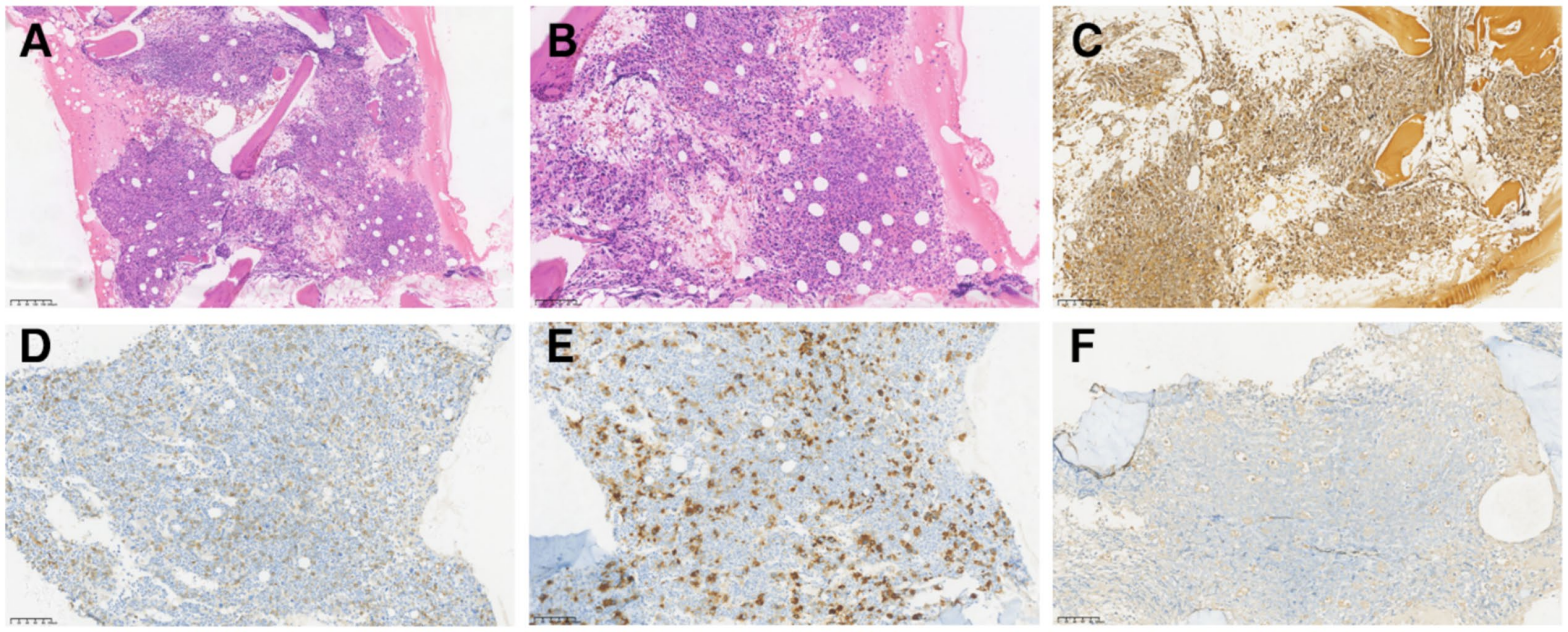
Microscopic image of histological analysis and immunohistochemistry of AMKL patients. **(A)** Myelodysplasia is highly active, with a significant number of primitive immature cell infiltration (≥20%), distributed in sheets or clusters. Normal hematopoietic tissue (such as red blood cells and granulocytes) was notably reduced. HE, 10×; **(B)** The primitive cells vary in size, with round or oval nuclei, fine chromatin, distinct nucleoli, and small and basophilic cytoplasm. Immature megakaryocytes are observable. HE, 20×; **(C)** Grade MF-2, Gomori silver staining, 20×; **(D)** Positive cell membrane of the primitive cell for CD34, 20×; **(E)** Positive cytoplasm of the primitive cells for CD61, 20×; **(F)** Negative expression of MPO in primitive cells, 20×.

Pathological biopsy revealed that myelodysplasia is present, featuring an increase in the number of primitive cells, distributed in sheets or clusters, and dysplastic megakaryocytes (A, B). The results of reticular fiber staining indicated moderate fibrous tissue hyperplasia (C). Immunohistochemical results demonstrated that CD34 (D) and CD61 (E) were expressed in the naive cells within the bone marrow tissue, while MPO was negatively expressed (F). The histopathological and immunohistochemical staining results of AMKL patients are presented in Figure 4.

### Immunophenotyping features

The results of bone marrow flow cytometric immunophenotyping in 21 cases of 23 AMKL patients were available for analysis. The patients who underwent flow cytometric immunotyping were divided into two groups of pediatric patients and adults for classified analysis and display, consisting of 10 children and 11 adults. The characteristic parameters of bone marrow cell flow immunotyping of AMKL patients in this study are depicted in Table 2.

**Table 2:** Flow cytometric immunophenotyping features of the patients with AMKL.

Immunophenotyping indexes	Pediatric patients (*n* = 10)	Adult patients (*n* = 11)
CD41	10 (100)	10 (90.91)
CD42	9 (90)	11 (100)
CD61	9 (90)	10 (90.91)
CD13	5 (50)	8 (72.73)
CD33	6 (60)	10 (90.91)
CD34	8 (80)	9 (81.82)
HLA-DR	4 (40)	4 (36.36)
CD117	7 (70)	9 (81.82)
CD56	5 (50)	7 (63.64)
CD3	4 (40)	3 (27.27)
CD4	3 (30)	5 (45.45)
CD7	4 (40)	4 (36.36)

CD, leukocyte differentiation antigen; HLA-DR, human leukocyte Antigen; CD41, CD42 and CD61 were megakaryon antigens, CD34 and HLA-DR were hematopoietic progenitor cell antigens, CD13, CD33 and CD117 were myeloid antigens, CD3, CD4 and CD7 are T cell antigens (AMKL can be abnormally expressed), and CD56 is NK cell antigen.

### Analysis of cytogenetic and molecular biological assay

The results of cytogenetics and molecular biology detection in 21 cases of all 23 cases of AMKL patients were available for analysis. Therefore, the 21 patients who received cytogenetic and molecular biological tests were classified into two groups of pediatric patients and adults for classified analysis and presentation, including 10 children and 11 adults. The parameters of cytogenetic and molecular biological characteristics of AMKL patients are shown in Table 3.

**Table 3:** Analysis of karyotype and molecular biology assay of the patients with AMKL.

Indexes	Pediatric patients (*n* = 10)	Adult patients (*n* = 11)
Cytogenetic (chromosome and karyotype) assay		
Normal karyotypes	2 (20)	2 (18.18)
Complex karyotypes	7 (70)	6 (54.55)
+21	7 (70)	5 (45.45)
+6	3 (30)	4 (36.36)
+8	4 (40)	3 (27.27)
+19	5 (50)	4 (36.36)
Molecular biological detection		
WT1	5 (50)	7 (63.64)
EVI1	6 (60)	4 (36.36)
TP53	3 (30)	3 (27.27)
CEBPA	2 (20)	3 (27.27)
GATA1	3 (30)	1 (9.09)
c-kit	1 (10)	2 (18.18)
ASXL1	2 (20)	3 (27.27)
TET2	0 (0)	2 (18.18)
MLL/AF9	1 (10)	0 (0)
DNMT3A	0 (0)	1 (9.09)
CSF3R	0 (0)	1 (9.09)

WT1, Wilms’ tumor gene 1; EVI1, ecotropic viral integration site 1; TP53, tumor protein p53; CEBPA, CCAAT/ enhancer Binding Protein α; GATA1, globin transcription factor 1; c-kit, c-kit proto-oncogene protein (stem cell factor receptor gene); ASXL1, additional sex combs-like 1; TET2, tet methylcytosine dioxygenase 2; DNMT3A, DNA methyltransferase 3α; CSF3R, colony stimulating factor 3 receptor.

### Comprehensive efficacy and prognostic evaluation

Among the 23 patients with AMKL encompassed in this study, two disheartened patients forsook treatment subsequent to the initial diagnosis of AMKL via bone marrow morphology. The remaining 21 patients underwent clinical intervention and treatment, and we procured adequate data related to clinical treatment. As depicted in Figure 4, among the 21 patients who underwent clinical treatment measures, 1 patient (4.76%) died after 1 course of chemotherapy, 3 patients (14.29%) succumbed to severe infection occasioned by bone marrow suppression after 2 courses of chemotherapy, and 7 patients (33.33%) achieved CR after 1 course of chemotherapy, 4 patients (19.05%) attained CR after 2 courses of chemotherapy, and 6 patients (28.57%) failed to achieve remission (NR) after 2 courses of induction chemotherapy. Correspondingly, a total of 6 patients received allogeneic hematopoietic stem cell transplantation (HSCT) in this study, among which 3 patients received HSCT after CR in the first induction chemotherapy, 1 patient received HSCT after CR in the second round of induction chemotherapy, and 2 patients with NR after induction chemotherapy underwent HSCT.

**Figure 5:**
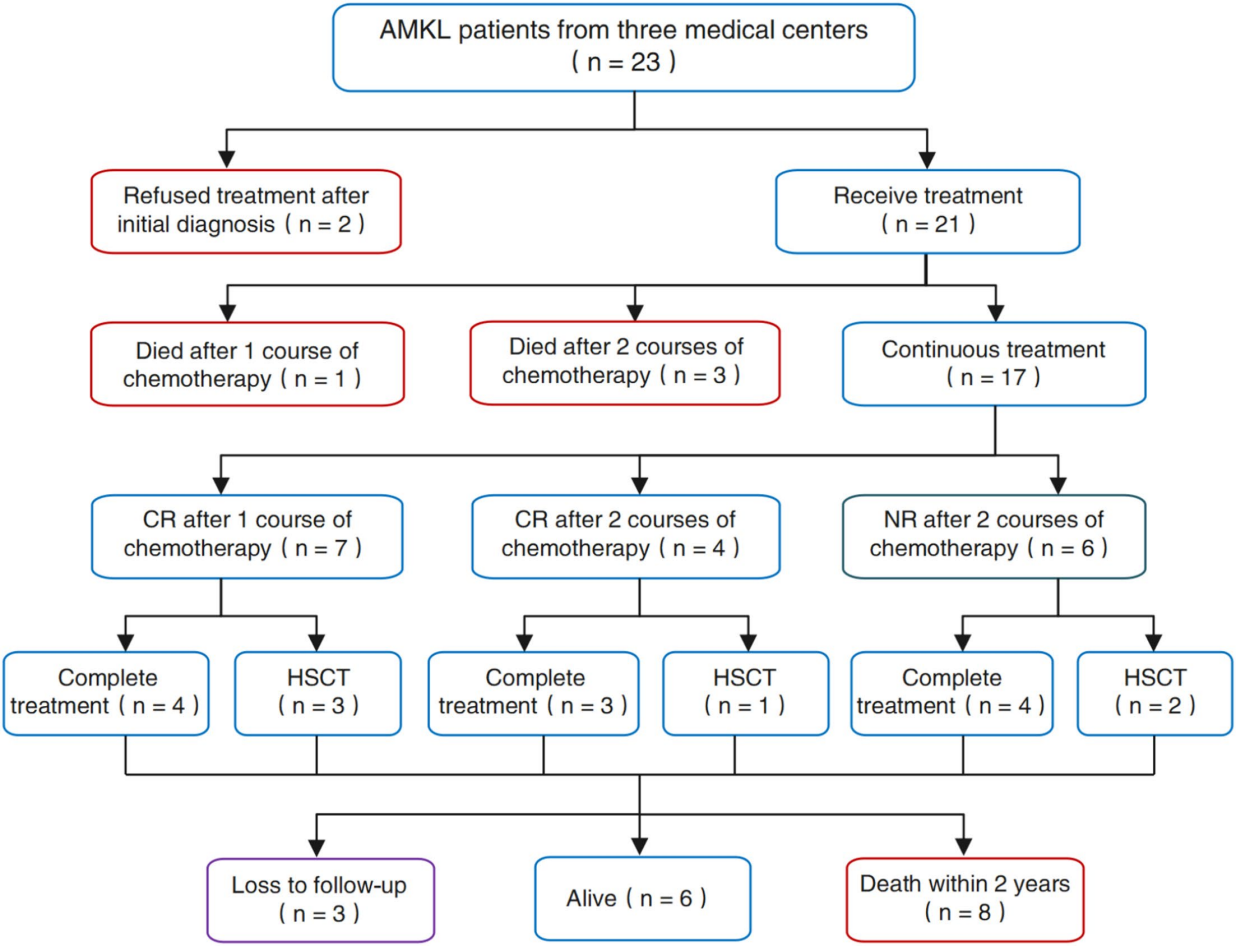
The comprehensive efficacy and outcomes of the patients with AMKL in our study.

We conducted follow-up on patients’ return to the hospital for review and hospitalization by utilizing an electronic inpatient record system, as well as through telephone follow-up until July 31, 2024. We discovered that among the 17 patients who received complete and standardized treatment and survived, 3 (17.65%) patients were lost to follow-up and 8 (47.06%) patients died within 2 years due to treatment failure attributed to disease progression, recurrence, and uncontrollable disease. The remaining 6 patients (35.29%) are still alive at present and have not experienced disease progression or recurrence. The comprehensive efficacy and prognostic outcomes of AMKL patients in this study are shown in Figure 5.

### Long-term survival analysis

**Figure 6:**
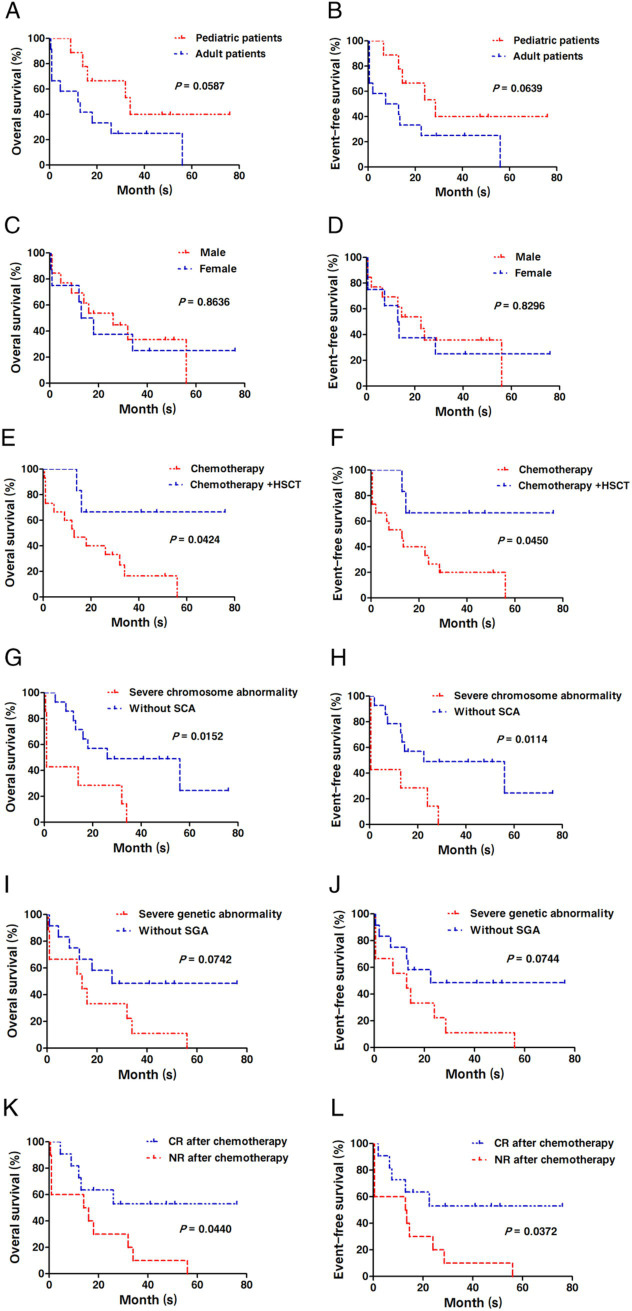
Comparative analysis of OS and EFS after AMKL patients in this study were grouped differently. **(A,B)** Comparison of OS and EFS of AMKL patients in the pediatric group and the adult group. (C-D) Comparison of OS and EFS in AMKL patients of different gender groups. **(E,F)** Comparison of OS and EFS in AMKL patients treated with chemotherapy alone and chemotherapy combined with HSCT. **(G,H)** Comparison of OS and EFS in AMKL patients with or without severely cytogenetic abnormalities. **(I,J)** Comparison of OS and EFS in AMKL patients with or without severely abnormal molecular biological results. **(K,L)** Comparison of OS and EFS in AMKL patients with or without CR after 1 ~ 2 courses of induction chemotherapy.

The median follow-up period was 33.5 months (ranging from 4.5 to 76 months) as of July 31, 2024. As illustrated in Figure 6, all AMKL patients included in this study were categorized and analyzed for survival in accordance with patient category (child or adult), gender, treatment modality (chemotherapy alone or combined with HSCT), cytogenetic detection results (whether complex karyotype or with abnormal chromosomes), molecular biological detection results, and feedback on the efficacy of initial induction chemotherapy. The results of the survival analysis indicated that: The OS and EFS of AMKL patients treated with chemotherapy alone were inferior to those treated with chemotherapy combined with HSCT (all *p* < 0.05). Additionally, AMKL patients with severely abnormal cytogenetic test results had poorer OS and EFS (all *p* < 0.05). Concurrently, the OS and EFS of AMKL patients who achieved CR after 2 courses of induction chemotherapy were significantly superior to those of AMKL patients who did not achieve CR (all p < 0.05). Nevertheless, there was no statistical significance in OS and EFS among different groups (pediatric group and adult group), different gender groups, and AMKL patients with or without molecular biological abnormalities (all *p* > 0.05).

## Discussion

AMKL is a highly heterogeneous form of AML that originates from hematopoietic stem cells and is characterized by impaired differentiation due to excessive and aberrant proliferation of immature megakaryocytes (12), accounting for approximately 1% of all AML types (13), which was initially reported by Von Boros in 1931 (14). In the subsequent decades, the clinical rarity of AMKL and the absence of accurate and reliable diagnostic criteria contributed to its low diagnosis rate. In 1978, Breton Gorius et al. (15) employed electron microscope technology and platelet peroxidase (PPO) to assist in the diagnosis of AMKL, thereby enhancing the diagnostic accuracy of AMKL to a certain extent. In 1985, AMKL was officially incorporated into the AML-M7 category of FAB (16), and in 2008, WHO formulated the precise diagnostic criteria for AMKL, that is, ≥20% of bone marrow original cells and >50% of bone marrow original cells are derived from megakaryocytes, or platelet-specific antigens, such as factor VII, CD41, CD42, and CD61, which could be detected through bone marrow puncture biopsy (17).

Due to the low incidence and dismal prognosis of AMKL, patients afflicted with this malignant disorder typically seek medical attention with a constellation of non-specific manifestations or symptoms, such as pallid skin mucosa, asthenia, fever, bleeding gums or skin, hepatosplenomegaly, lymph node enlargement (18). Some studies posit that the pathogenic mechanism of AMKL is likely the unrestrained proliferation of bone marrow immature megakaryocytes, which subsequently inhibits normal hematopoiesis and function (19). Therefore, in general, AMKL patients are characterized by pancytopenia, particularly reduced platelet counts, which can engender confusion in clinical diagnosis, as numerous hematological diseases exhibit these non-specific symptoms or signs. In this study, nearly all patients presented with abnormally decreased hemoglobin and platelet levels, in line with previous studies (18, 19).

It is a well-established fact that DS is the most prevalent chromosomal disorder in humans, and DS is closely associated with an elevated risk of leukemia. Notably, the incidence of AMKL in DS patients is up to 200 times higher than that in healthy individuals. Transient abnormal myelopoiesis (TAM) occurs in 10–20% of DS infants, and TAM is an essential step in the progression to AMKL (20, 21), meaning that TAM may rapidly progress to AMKL or resolve spontaneously within 3 months after onset, while AMKL may recur within 3 years after resolution (22). Nevertheless, AMKL has an exceptionally favorable prognosis in DS and a significantly better response to chemotherapy compared to traditional childhood myeloid leukemia (20, 23). In reality, the overall survival rate for patients with DS combined with AMKL is currently approximately 10–30% (20, 24). Although most cases of AMKL occur within the first few months of life, current research indicates that AMKL might be a crucial cause of maternal stillbirth and fetal death in utero (20, 25). Interestingly, among the AMKL patients included in this study, 7 patients had additional copies of chromosome 21, and 3 patients had GATA1 mutations. Therefore, we consulted clinical experts in the genetics laboratory to address this confusion, and it was discovered that such patients carried abnormal chromosomes and gene mutations. However, there was no clear evidence-based medical evidence to confirm their Down syndrome, nor were there clinical manifestations and morbidity, so our team identified the patients included in the study as non-DS-AMKL, subsequently carried out standard induction remission chemotherapy for them, and recommended bone marrow transplantation at the first remission.

Bone marrow biopsy results frequently indicate abnormal megakaryocyte proliferation and extensive myelofibrosis, which is prone to being overlooked and misdiagnosed clinically. In this study, all AMKL patients underwent bone marrow puncture biopsy. Among them, 16 cases (69.57%) encountered difficulties in obtaining the sample when completing bone marrow puncture upon admission, namely, “bone marrow dry aspiration (tap),” which was largely consistent with previous research findings (26, 27). Besides, the observation of megakaryocyte ultrastructure and detection of PPO positive reaction via electron microscopy are conducive to the diagnosis of AMKL (25, 28).

AMKL possesses a complex cytogenetic profile and is more prevalent in children with DS, and it constitutes the most common malignant hematologic disease in DS children (5). The genetic and molecular biological characteristics of DS-AMKL encompass trisomy 21 (T21) and GATA1 mutations, accompanied by chromatin regulatory factors such as the cohesive protein subunit EZH2 or signaling molecules such as JAK/STAT and RAS pathways (29, 30). However, non-DS-AMKL typically does not carry GATA1 mutations however is prone to cytogenetic chromosomal translocation, which gives rise to the expression of related oncogenic driver genes or fusion proteins, resulting in a significantly poorer prognosis for non-DS-AMKL patients compared to DS-AMKL patients (31). Among the patients who underwent cytogenetic and molecular biological tests in this study, with the exception of 4 patients who were normal, the remainder of the patients exhibited chromosomal karyotype abnormalities, mutations, or fusion genes, suggesting that AMKL patients are prone to cytogenetic chromosomal aberrations, albeit without regularity.

Owing to the signs and symptoms of AMKL that bear resemblance to those of numerous myeloid tumors. Hence, it is of paramount importance to accurately diagnose and treat AMKL, which has a dismal prognosis. The differential diagnosis of AMKL mainly lies in histopathology, encompassing MDS 5q-, RARST (now MDS/MPN with SF3B1 mutation and thrombocytosis), panmyelofibrosis, and myeloproliferative tumors (including evolving cases). AML with defining genetic abnormalities, especially AML with RBM15::MRTFA fusion and AML with MECOM rearrangement, and other entities/types of AML defined by differentiation. Of note, chimeric fusion genes (such as CBFA2T3::GLIS2, NUP98::KDM5A, and RBM15::MRTFA) have been demonstrated to exert an influence on the prognosis of children with AMKL.

Specifically, the CBFA2T3-GLIS2 fusion gene is the outcome of recessive inversion of chromosome 16. AMKL is the most prevalent acute myeloid leukemia (AML) in children. Studies have indicated that CBFA2T3::GLIS2 is the most common chimeric oncogene in non-DS-AMKL, representing the highly aggressive nature of leukemia (32). It is closely linked to adverse circumstances such as poor therapeutic efficacy and prognosis, high recurrence rate, and short survival (33, 34). Researches by relevant scholars have revealed that CBFA2T3-GLIS2 exerts downstream effects through fusion binding and upregulated transcription factor gene network, resulting in dysregulation of developmental pathway signals including NOTCH, Hedgehog, TGFβ and WNT, thereby mediating the occurrence and progression of AMKL. Simultaneously, the sensitivity of AMKL cells to targeted inhibitors has decreased (3, 35). Targeting the CBFA2T3-GLIS2 fusion gene can enhance the prognosis of AMKL, which is conducive to long-term prognosis and effectively prolongs survival (36–38). Therefore, the CBFA2T3-GLIS2 fusion gene constitutes an extremely crucial therapeutic target for AMKL. At the same time, NUP98::KDM5A is also intimately associated with a poor prognosis and short survival in AMKL patients. After establishing an AMKL disease model with overexpression of NUP98-KDM5A, it is discovered that NUP98-KDM5A can effectively impede cell differentiation and maturation. Its expression is correlated with novel markers such as SELP, MPIG6B and NEO1, and anomalously regulates the activation of the JAK–STAT signaling pathway. Nevertheless, the AMKL model of NUP98-KDM5A shows susceptibility to *in vitro* treatment with the clinically approved JAK2 inhibitor Ruxolitinib. This is likely to represent a potential breakthrough for the treatment of AMKL in the future (39). RBM15::MRTFA is an intriguing entity in AMKL patients, and it is more common in female patients. AMKL patients accompanied by RBM15::MRTFA are highly likely to be complicated with liver fibrosis, and these patients are almost all female (40). One study (41) evaluated morphology, cytogenetics, and genome sequencing in 107 non-DS AMKL patients. Different fusion genes such as RBM15::MRTFA (20%), CBFA2T3::GLIS2 (16%), NUP98 (10%), KMT2A (7%), TEC::MLLT10 (2%), MECOM (1%) and FUS::ERG (1%) were identified, disappointingly, AMKL patients with CBFA2T3::GLIS2 or KMT2A rearrangement and NUP98 fusion gene have a poorer prognosis than other patients, while those with RBM15::MRTFA have a better prognosis, which will assist in risk stratification and treatment selection for AMKL patients.

It is notable that AMKL is a nonspecific malignant hematological disorder without specific cytogenetic and molecular biological abnormalities. Relevant studies have verified that AMKL can transform from other hematological diseases (42, 43), and a very small number of patients with AMKL can develop treatment-related AMKL secondary to the treatment of hematological tumors (44). It is proposed that patients’ past hematological history and treatment history should be given attention in clinical work to prevent missed diagnoses or misdiagnoses. Although significant progress has been made in the diagnosis of AMKL in recent years, it is disheartening that no breakthrough has been achieved in the treatment of AMKL, and it remains a malignant hematologic disease with a low long-term survival rate and poor prognosis. In clinical practice, the treatment of AMKL using conventional standard induction chemotherapy yields poor outcomes, a low remission rate, and a high recurrence rate. Currently, there is no recognized, unified, and effective treatment modality and regimen in the academic field (31, 45).

Among the 23 patients with AMKL encompassed in this study, 21 patients underwent clinical intervention and treatment, and 1 patient died after 1 course of chemotherapy, 3 patients died after 2 courses of chemotherapy. While 7 patients achieved CR after 1 course of chemotherapy, 4 patients attained CR after 2 courses of chemotherapy, and 6 patients failed to achieve CR after 2 courses of induction chemotherapy. We conducted follow-up research until July 31, 2024, and discovered that only 6 patients are still alive, the remaining patients died due to recurrence, uncontrolled progression or ineffective treatment. We found that the OS and EFS of AMKL patients treated with chemotherapy alone were inferior to those treated with chemotherapy combined with HSCT, and the OS and EFS of AMKL patients who achieved CR after 2 courses of induction chemotherapy were significantly better than those who did not achieve CR.

Studies have demonstrated that epigenetic abnormalities and special target gene alterations play a crucial role in AMKL, and the combination of epigenetic drugs and targeted therapy could also benefit patients (46, 47). Additionally, other studies have discovered that Ara-C combined with anthracyclines can achieve CR in nearly 50% of AMKL patients, and hematopoietic stem cell transplantation after the first CR can enhance the survival rate of patients (48, 49). The results of this study are essentially consistent with the aforementioned conclusions.

Indeed, the detection of chimeric fusion genes through transcriptome RNA sequencing, while personalized disease risk or disease risk stratification targeting cytogenetics and molecular biology, will hold significant practical significance for the treatment and prognosis of AMKL patients in clinical practice. Haploid hematopoietic stem cell transplantation and post-transplantation cyclophosphamide-based induced remission may enhance the survival rate of AMKL children with fusion genes (50). In addition, most studies still contend that HSCT should be conducted as early as possible to achieve the first remission after induced remitting chemotherapy as the optimal strategy to prevent AMKL recurrence (51, 52), and targeted therapy targeting special chimeric fusion genes or AMKL-specific surface antigens can also improve the prognosis of AMKL patients (4–6, 53). It should be noted that currently, MRD detection during the course of AMKL needs to be emphasized, as it is an important tool for evaluating the treatment effect and prognostic risk (54).

## Conclusion

In conclusion, AMKL is a clinically rare leukemia, the patients should be encouraged to participate in clinical new drug research, and measurable residual disease (MRD) should be closely monitored, and treatment methods should be dynamically adjusted according to patient tolerance, genetic risk stratification, and post-treatment MRD. Certainly, in addition to standard induction chemotherapy, the novel targeted therapy can also be incorporated, and HSCT should be conducted as soon as possible upon achieving the first CR to maximize the prognosis of patients. More in-depth exploration and research should be actively pursued from the perspective of molecular biological targets or genetics in the future, in order to provide new references and guidance for the clinical diagnosis and treatment of AMKL, basic research, and clarification of pathogenic mechanisms.

## Data Availability

The original contributions presented in the study are included in the article/supplementary material, further inquiries can be directed to the corresponding author/s.
